# Sub-Acute Treatment of Curcumin Derivative J147 Ameliorates Depression-Like Behavior Through 5-HT_1A_-Mediated cAMP Signaling

**DOI:** 10.3389/fnins.2020.00701

**Published:** 2020-07-08

**Authors:** Jianxin Li, Ling Chen, Gaowen Li, Xiaojuan Chen, Sisi Hu, Liang Zheng, Victor Luria, Jinpeng Lv, Yindi Sun, Ying Xu, Yingcong Yu

**Affiliations:** ^1^Department of Gastroenterology, Wenzhou No. 3 Clinical Institute Affiliated to Wenzhou Medical University, Wenzhou People’s Hospital, Wenzhou, China; ^2^Department of Clinical Pharmacology, Key Laboratory of Clinical Cancer Pharmacology and Toxicology Research of Zhejiang Province, Affiliated Hangzhou First People’s Hospital, Zhejiang University School of Medicine, Hangzhou, China; ^3^Ningbo College of Health Sciences, Ningbo, China; ^4^Department of Pharmaceutical Sciences, School of Pharmacy & Pharmaceutical Sciences, University at Buffalo, The State University of New York, Buffalo, NY, United States; ^5^Department of Systems Biology, Harvard Medical School, Boston, MA, United States; ^6^College of Pharmaceutical Engineering and Life Sciences, Changzhou University, Changzhou, China; ^7^Department of Traditional Medical Orthopedics, Honghui Hospital, Xi’an Jiaotong University, Xi’an, China

**Keywords:** J147, antidepressant-like effects, 5-HT_1A_, 5-HT_1B_, cAMP/PKA, BDNF

## Abstract

**Background:**

Major depressive disorder (MDD) is a severe mental disorder related to the deficiency of monoamine neurotransmitters, particularly to abnormalities of 5-HT (5-hydroxytryptamine, serotonin) and its receptors. Our previous study suggested that acute treatment with a novel curcumin derivative J147 exhibited antidepressant-like effects by increasing brain derived neurotrophic factor (BDNF) level in the hippocampus of mice. The present study expanded upon our previous findings and investigated the antidepressant-like effects of sub-acute treatment of J147 for 3 days in male ICR mice and its possible relevancy to 5-HT_1A_ and 5-HT_1B_ receptors and downstream cAMP-BDNF signaling.

**Methods:**

J147 at doses of 1, 3, and 9 mg/kg (via gavage) was administered for 3 days, and the anti-immobility time in the forced swimming and tail suspension tests (FST and TST) was recorded. The radioligand binding assay was used to determine the affinity of J147 to 5-HT_1A_ and 5-HT_1B_ receptor. Moreover, 5-HT_1A_ or 5-HT_1B_ agonist or its antagonist was used to determine which 5-HT receptor subtype is involved in the antidepressant-like effects of J147. The downstream signaling molecules such as cAMP, PKA, pCREB, and BDNF were also measured to determine the mechanism of action.

**Results:**

The results demonstrated that sub-acute treatment of J147 remarkably decreased the immobility time in both the FST and TST in a dose-dependent manner. J147 displayed high affinity *in vitro* to 5-HT_1A_ receptor prepared from mice cortical tissue and was less potent at 5-HT_1B_ receptor. These effects of J147 were blocked by pretreatment with a 5-HT_1A_ antagonist NAD-299 and enhanced by a 5-HT_1A_ agonist 8-OH-DPAT. However, 5-HT_1B_ receptor antagonist NAS-181 did not appreciably alter the effects of J147 on depression-like behaviors. Moreover, pretreatment with NAD-299 blocked J147-induced increases in cAMP, PKA, pCREB, and BDNF expression in the hippocampus, while 8-OH-DPAT enhanced the effects of J147 on these proteins’ expression.

**Conclusion:**

The results suggest that J147 induces rapid antidepressant-like effects during a 3-day treatment period without inducing drug tolerance. These effects might be mediated by 5-HT_1A_-dependent cAMP/PKA/pCREB/BDNF signaling.

## Introduction

Major depressive disorder (MDD) is a stress-related mental disorder related to the deficiency of monoamine neurotransmitters, particularly to 5-HT (5-hydroxytryptamine, serotonin) and its receptors (Kennedy et al., 2002; Kessler et al., 2003; Patel et al., 2007). The link between depression and serotonin is supported by studies suggesting that most antidepressants may increase serotonin levels, such as the serotonin transporter inhibitors (SERTIs), the dual serotonin and norepinephrine reuptake inhibitors (SNRIs) and the selective serotonin reuptake inhibitors (SSRIs) (Chen et al., 2011). However, both SNRIs and SSRIs induce delayed antidepressant response and undesired side effects, which substantially hamper their clinical application.

Serotonin receptors 1A (5-HT_1A_), 1B (5-HT_1B_), and 7 (5-HT_7_) receptors play a vital role in the pathophysiology of depression. Pre- and post-synaptic 5-HT_1A_ receptor and 5-HT_1B_ receptor play opposite roles in depression (Pucadyil et al., 2005; Albert, 2012; Artigas, 2013). The activation of pre-synaptic 5-HT_1A_ receptor (5-HT_1A_R, an auto-receptor) induces activation of hyperpolarizing K^+^ channels and inhibition of neuronal activity (Richardson-Jones et al., 2010; Vahid-Ansari et al., 2017), and 5-HT_1B_ autoreceptor exhibits negative feedback by inhibiting serotonergic activity (Marta et al., 2016). By contrast, the activation of post-synaptic 5-HT_1A_ and 5-HT_1B_ receptors enhances the neuronal activation by upregulating multiple signaling molecules, such as cyclic adenosine monophosphate (cAMP), cyclic-AMP dependent protein kinase A (PKA), cAMP response element binding protein (CREB), and brain derived neurotrophic factor (BDNF) (Zheng et al., 2017). Currently, clinical use of SSRIs significantly increases extra-neuronal serotonin (Samuels et al., 2015), which in turn desensitizes pre-synaptic 5-HT_1A_ receptors and activates post-synaptic 5-HT_1A_ receptors (Albert and Lemonde, 2004), leading to antidepressant-like effects. The 5-HT_7_ receptor stimulates cAMP formation by activating adenylyl cyclases (AC) (Norum et al., 2003). In addition, 5-HT_7_ receptors are known to form heterodimers with 5-HT_1A_ receptors (Renner et al., 2012) and this heterodimer modulates cAMP production. Post-synaptic 5-HT_1B_ receptors are co-localized with N-methyl-D-aspartate (NMDA) receptors on dendrites. The antidepressant effects of SSRIs may be mediated by a signaling interaction between 2-amino-3-hydroxy-5-methyl-4-isoxazolepropionate (AMPA) receptors and 5-HT_1B_ receptors (Cai et al., 2013). Thus, 5-HT_1B_ receptor’s activation is seemingly necessary for treating depression (O’Neill and Conway, 2001).

Curcumin is a turmeric component that elicits antioxidant, anti-infiammatory, and antidepressant-like effects (Xu et al., 2005a, b, 2006). However, due to its poor bioavailability, the application of curcumin for treatment of depression is limited. To circumvent this problem, curcumin derivative J147 is identified as a potent neurotrophic compound whose stability and bioavailability are greater than those of curcumin. According to multiple neurochemical assays, J147 possesses the neurotrophic activities that curcumin lacks (O’Neill and Conway, 2001). Recent studies suggested that J147 ameliorates cognitive impairment in a mouse model of Alzheimer’s disease (AD) (Chen et al., 2011). Our previous study suggested that acute administration of J147 via gavage produces antidepressant-like effects dose-dependently, peaking at 1 h after treatment, which may be involved in 5-HT_1A_ receptor or partially involved in 5-HT_1B_ receptor (Lian et al., 2018). The present work expanded upon the previous study by examining the antidepressant-like effects of sub-acute treatment of J147 for 3 days in the forced swimming and tail suspension tests (FST and TST). The radioligand binding assay was used to determine the affinity of J147 to 5-HT_1A_ and 5-HT_1B_ receptor. The 5-HT_1A_ or 5-HT_1B_ receptor agonist and their antagonists were used to determine whether the effect of J147 on depression is related to 5-HT_1A_ or 5-HT_1B_ receptor dependent signaling.

## Materials and Methods

### Animals and Housing

Adult male ICR mice (22–30 g) were obtained from the Animal Center of University at Buffalo, the State University of New York, and Wenzhou Medical University Animal center. Water and food were freely available in the animals’ home cages. Mice were kept in a temperature-controlled room under standard laboratory conditions, with a light/dark cycle (12:12 h, lights on at 6:00 a.m.), constant temperature (22 ± 2°C), and humidity (55 ± 10%). All procedures in this study followed the “NIH Guide for the Care and Use of Laboratory Animals” (revised 2011) and were reviewed and approved by the Animal Care and Use Committee of the State University of New York at Buffalo and Wenzhou Medical University.

### Chemicals and Drug Administration

J147 (purity: ≥99% by HPLC) was kindly provided by Dr. David Schubert at Salk Institute, California, United States. It has the medicinal chemical properties of a good central nervous system lead compound with respect to size (351 MW), cLogP (4.5), total polar surface area (41.9), and ideal pharmacokinetics (Chen et al., 2011). J147 was dissolved in a vehicle that consisted of 5% dimethyl sulfoxide (DMSO, Sigma Chemical Co., United States), 5% polyethylene glycol 660 hydroxystearate (HS15, Sigma Chemical Co., United States), and 90% saline on the day of the experiment. The final concentrations of J147 were 0.1 mg/ml, 0.3 mg/ml, and 0.9 mg/ml. Imipramine (Sigma Chemical Co., United States), NAD-299 (a selective 5-HT_1A_ receptor antagonist), NAS-181 (a selective 5-HT_1B_ receptor antagonist), and 8-OH-DPAT (a selective 5-HT_1A_ receptor agonist) were obtained from Bio-Techne Corporation (Minneapolis, MN, United States) and dissolved in saline. The cAMP and PKA ELISA kits were purchased from Enzo Life Sciences (United States). The primary antibodies of anti-pCREB, anti-BDNF, and all the secondary antibodies were purchased from Abcam (Cambridge, MA, United States).

Considering that J147 was easily absorbed via gavage (i.g.) in our previous study, mice were given J147 (1, 3, 9 mg/kg, i.g.) or imipramine (10 mg/kg, i.p.) once a day for 3 days. The behavioral tests were performed 1 h (J147) or 30 min (imipramine) after last treatment. To investigate whether 5-HT_1A_ or 5-HT_1B_ receptors mediate the effects of J147, mice were pre-treated with NAD-299, NAS-181 or 8-OH-DPAT by intraperitoneal injection at a dose of 0.5 or 1 mg/kg, 30 min prior to the J147 or imipramine administration. To investigate the interaction of J147 and 5-HT_1A_ or 5-HT_1B_ receptors, the minimum dose of J147 was given together with the 5-HT_1A_ receptor agonist 8-OH-DPAT and the maximum dose was given together with the receptor antagonists NAD-299 and NAS-181.

One cohort of mice (10 mice/group) was assessed for depression-like behaviors in the tail suspension and locomotor activity (LMA) tests. The mouse hippocampus was taken for immunoblot analyses after the behavioral tests. The other cohort of mouse was subject to the FST (10 mice/group) before undergoing hippocampus extraction for the enzyme-linked immunosorbent assay (ELISA) test.

### Forced Swimming Test

Mice were individually placed in a transparent tank (height: 25 centimeters (cm), diameter: 10 cm) for 15 min (pre-swim session), a tank which was filled with water (23–25°C) 10 cm deep. Mice were dropped in the tank again for 6 min after 24 h elapsed (test session). The immobility time was recorded in the last 4 min of the test session. A mouse was deemed immobile when it stops struggling and floats motionlessly in the water, apart from tiny movements which are necessary to keep its head above the water.

### Tail Suspension Test

Mice were suspended by an adhesive tape and affixed approximately 1 cm from the tip of the tail, 50 cm above the floor. Each mouse was hung for a test period of 6 min, and the duration of immobility was recorded during the last 4 min of the test period.

### Locomotor Activity

The floor of open field chamber was divided into nine equal squares. Each mouse was placed in the center of the chamber and allowed to explore for 15 min freely, during which the number of line crossings (when all four paws cross the line into a new square) was recorded. The cross count was recorded during the last 10 min of each trial.

### Affinities of J147 to 5-HT_1A_ and 5-HT_1B_ Receptors

Male ICR mice were killed by cervical dislocation, and the frontal cortex was dissected and homogenized in 40 volumes of ice-cold buffer (50 mM Tris-HCl buffer pH 7.4). The homogenates were centrifuged at 40,000 × g for 10 min at 4°C. The pellet was gently resuspended and centrifuged again. Membranes prepared in this manner could be stored at −80°C for up to 1 week. To assess the binding affinity of J147 to 5-HT_1A_ and 5-HT_1B_ receptor from mice frontal cortex, competitive binding assays were performed as previously described (Peroutka and McCarthy, 1989; Gozlan et al., 1995). The radioactivity was determined by liquid scintillation counting. The binding assays were performed in duplicate in three independent experiments.

### Measurement of cAMP and PKA Levels in the Hippocampus by ELISA

Cyclic adenosine monophosphate and cyclic-AMP dependent PKA levels in the hippocampus were measured with mouse cAMP and PKA ELISA kits respectively according to the manufacturer’s instructions (R&D Systems Inc., Minneapolis, MN, United States). The total protein concentration in the hippocampus was determined using the Coomassie (Bradford) colorimetric assay. Absorbance values were read at 505 nm using a microplate reader (SpectraMax, CA, United States).

### Immunoblot Analysis

Mice were sacrificed immediately after behavioral tests. Hippocampal tissues were dissected and immediately stored at −80°C. The total concentrations of proteins were measured using the BCA assay kit (Thermo Fisher Scientific, United States). They were then thawed and subsequently homogenized in RIPA lysis buffer containing protease and phosphatase inhibitors and centrifuged at 14,000 rpm for 20 min at 4°C for protein measurement. Samples (60 μg protein each) were separated using SDS-PAGE before transferring to PVDF membranes (0.20 μm; Millipore, Billerica, MA, United States). Nonspecific binding events were blocked with 5% skim milk for 90 min (Lv et al., 2020). Membranes were subsequently incubated with the appropriate primary antibodies for rabbit anti-5HT_1A_ receptor (at a dilution of 1:1000; Abcam, United States), anti-5HT_1B_ receptor (at a dilution of 1:1000; Abcam, United States), anti-5HT_7_ receptor (at a dilution of 1:1000; Abcam, United States), anti-pCREB (at a dilution of 1:1000; Abcam, United States), anti-BDNF (at a dilution of 1:1000; Abcam, United States), and anti-β-Actin (at a dilution of 1:5000; Abcam, United States) overnight at 4°C. After washing with TBST (0.1%) and incubation with secondary antibodies (the goat anti-rabbit IgG at a dilution of 1:5000; Santa Cruz, United States), ECL kit was used to visualize the immune complex by chemiluminescence. The specific bands were detected using Gel Doc XR System (Bio-Rad, United States) and quantified using Quantity One software.

### Statistical Analysis

Data were expressed as mean ± standard error of the mean (SEM). One-way analysis of variance (ANOVA) was used for multiple comparisons following a *post-hoc* Dunnett’s test or a *post-hoc* Tukey’s HSD test. For comparisons between two groups, data were analyzed by the student’s *t*-test. All statistical analyses were carried out using GraphPad Prism 5. The statistical level of significance was set to *p* < 0.05. The receptor binding and monoamine uptake data were analyzed using one-site nonlinear regression of concentration–effect curve. The Ki values were calculated using Cheng–Prusoff equation: Ki = IC50/[(L/Kd)+1], where the IC50, L, and Kd are the half maximal inhibitory concentration, the substrate concentration, and the dissociation constant of radioligand, respectively.

## Results

### J147 Reduced the Immobility Time in Forced Swimming and Tail Suspension Tests

**FIGURE 1:**
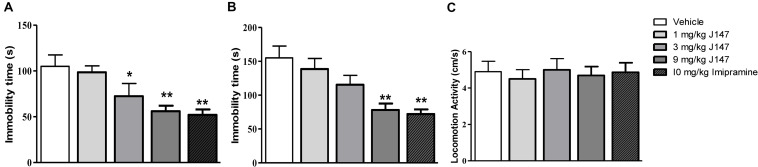
The effects of J147 on the duration of immobility in the forced swimming and tail suspension tests. The immobility time in the forced swimming and tail suspension tests was decreased after administration of J147 (3, 9 mg/kg, i.g) and imipramine (10 mg/kg, i.p) for 3 days **(A,B)**. Locomotor activity **(C)** did not change after treatment with drugs. The results represent the mean ± SEM, *n* = 10 per group. *p < 0.05, **p < 0.01, versus vehicle-treated group.

To evaluate the antidepressant-like effects of sub-acute J147 administration in mice, the immobility time in the FST and TST was recorded. As shown in Figures 1A,B, administration of J147 once a day for 3 days produced a dose-dependent antidepressant-like effect [*F*(3, 44) = 2.94, *p* < 0.05, Figure 1A; *F*(3, 44) = 3.71, *p* < 0.05, Figure 1B], i.e., J147 at doses of 3 and 9 mg/kg significantly reduced the immobility time in the FST (*p* < 0.05; *p* < 0.01), while high dose of J147 at 9 mg/kg also significantly reduced the immobility time in the TST (*p* < 0.01). The doses that induced the reduction of immobility time did not change LMA (Figure 1C), suggesting sub-acute treatment with J147 does not stimulate or inhibit the central nervous system. These effects were similar to those of the positive drug imipramine in both the FST and TST.

To evaluate whether sub-acute treatment with J147 affected 5-HT_1A_, 5-HT_1B_, and 5-HT_7_ receptors, we assessed the expression of these receptors in the hippocampus. The results in Supplementary Figures S1A,C showed that J147 increased 5-HT_1A_ and 5-HT_7_ receptor levels dose-dependently after drug treatment, when compared to vehicle-treated groups (*p* < 0.05). As shown in Supplementary Figure S1B, sub-treatment of J147 did not increase the 5-HT_1B_ receptor expression significantly in the hippocampus.

### Radioligand Binding Studies of J147

**FIGURE 2:**
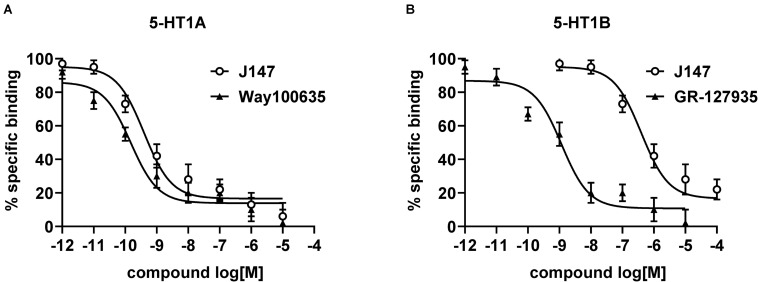
The affinity of J147 to 5-HT_1__*A*_ and 5-HT_1B_ receptor. The Ki values (nM) for inhibiting the binding of [^3^H]-8-OH-DPAT to 5-HT_1A_ **(A)** and 5-HT_1B_ **(B)** receptor by J147 and reference agents indicate that J147 bound to 5-HT_1A_ receptor with high affinity and J147 bound to 5-HT_1B_ receptor with low affinity. The Ki values (presented as mean ± SEM) were calculated from three independent experiments on different days, and each concentration was run in triplicate.

Radioligand binding assays were conducted to determine the affinity of J147 to mice 5-HT_1A_ and 5-HT_1B_ receptors. J147 showed high affinity to 5-HT_1A_ receptor and was less potent at 5-HT_1B_ receptor (Figure 2). The affinity constants (Ki) of J147 to 5-HT_1A_ receptors were compared with WAY-100635 under identical conditions in the same laboratory. WAY-100635 (Ki = 0.19 nM), a 5-HT_1A_ receptor full antagonist, was one order of magnitude greater than that of J147 (Ki = 0.42 nM). The affinity constants (Ki) of J147 to 5-HT_1B_ receptors were compared with GR-127935 under identical conditions in the same laboratory. GR-127935 (Ki = 1.29 nM), a 5-HT_1B_ receptor antagonist, was approximately three orders of magnitude greater than that of J147 (Ki = 612 nM).

### The Interaction of J147 With 5-HT_1A_ or 5-HT_1B_ Receptor in the FST and TST

**FIGURE 3:**
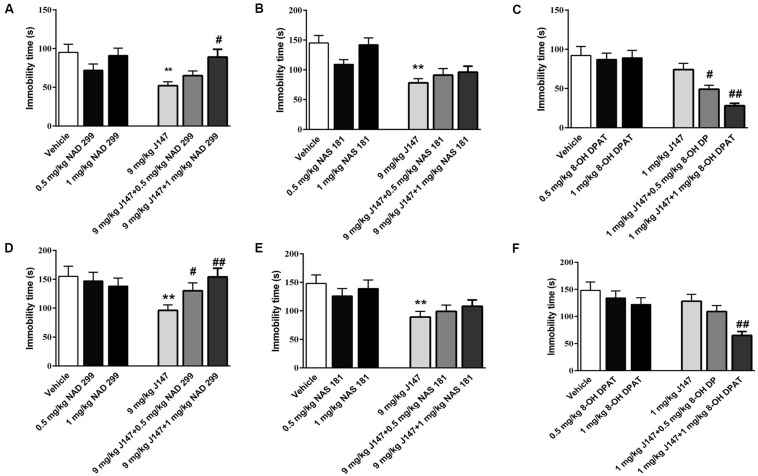
The interaction of J147 with 5-HT_1A_ or 5-HT_1B_ receptor in the forced swimming and tail suspension tests. Reduced immobility time induced by J147 in the forced swimming and tail suspension tests were reversed by the selective 5-HT_1A_ antagonist NAD-299 **(A,D)**. The selective 5-HT_1B_ antagonist NAS-181 did not affect J147’s effects on immobility time **(B,E)**. 5-HT_1A_ receptor agonist 8-OH-DPAT potentiated sub-threshold dose of J147 on the immobility time **(C,F)**. NAD-299, NAS-181 or 8-OH-DPAT used alone did not affect the immobility time. The results represent the mean ± SEM, *n* = 10 per group. **p < 0.01, versus vehicle-treated group; ^#^*p* < 0.05, ^##^*p* < 0.01, versus J147-treated group (9 mg/kg or 1 mg/kg).

To furthermore investigate whether 5-HT_1A_ or 5-HT_1B_ receptor influences the anti-immobility effects of J147, we administered 5-HT_1A_ receptor antagonist NAD-299, 5-HT_1B_ antagonist NAS-181 or 5-HT_1A_ receptor agonist 8-OH-DPAT, 30 min prior to J147 treatment. In the FST, pre-administered NAD-299 at dose of 1.0 mg/kg significantly reversed immobility time reduction induced by high dose J147 (9 mg/kg, i.g) as shown in Figure 3A (*p* < 0.01). On the other hand, NAS-181 failed to prevent such effects (Figure 3B). Moreover, 8-OH-DPAT at 0.5 and 1 mg/kg potentiated the effect of low dose J147 (1 mg/kg) on immobility time in a dose-dependent manner [*F*(2,33) = 5.64, *p* < 0.01, Figure 3C].

Similar results were obtained in the TST as shown in Figures 3D–F. The anti-immobility effects of J147 at 9 mg/kg (for 3 days) were reversed by 5-HT_1A_ receptor antagonist NAD-299 dose dependently [*F* (2, 33) = 4.41, *p* < 0.05, Figure 3D], while the 5-HT_1B_ receptor antagonist NAS-181 did not readily impact the effects of J147 (Figure 3E). Moreover, 5-HT_1A_ receptor agonist 8-OH-DPAT at dose of 1.0 mg/kg significantly potentiated the effects of low dose of J147 (1 mg/kg, i.g) on immobility time (*p* < 0.01, Figure 3F). However, the 5-HT_1A_ receptor antagonist NAD-299, 5-HT_1B_ receptor antagonist NAS-181, and 5-HT_1A_ receptor agonist 8-OH-DPAT used alone did not induce any change in immobility time in either the FST or the TST test (Figures 3D–F). These results suggested that there is an interaction between J147 and 5-HT_1A_ receptors, which contributes to J147-induced antidepressant-like behaviors.

### The Increased cAMP and PKA Levels in the Hippocampus Induced by J147 Were Reversed by a 5-HT_1A_ Receptor Antagonist NAD-299 Rather Than by 5-HT_1B_ Receptor Antagonist NAS-181

**FIGURE 4:**
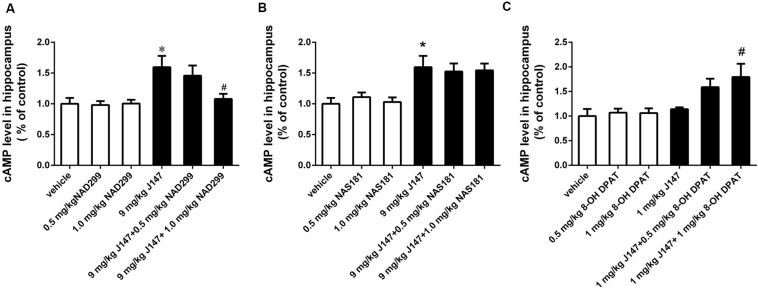
J147-induced increase in cAMP level in the hippocampus was reversed by the 5-HT_1A_ receptor antagonist NAD-299, but not 5-HT_1B_ receptor antagonist NAS-181. Mice received injections of NAD-299, NAS-181 or 8-OH-DPAT, 30 min prior to J147 for 3 days. J147 at dose of 9 mg/kg increased cAMP level in the hippocampus, which was reversed by 1 mg/kg NAD-299 **(A)** but not NAS-181 **(B)**. J147 at dose of 1 mg/kg did not change the level of cAMP, but its effect was potentiated by combination with 8-OH-DPAT (1 mg/kg) **(C)**. NAD-299, NAS-181, or 8-OH-DPAT used alone did not affect cAMP level, when compared to vehicle-treated groups. The results represent the mean ± SEM, *n* = 10 per group. *p < 0.05, versus vehicle-treated group; ^#^*p* < 0.05, versus J147-treated group (9 mg/kg or 1 mg/kg).

**FIGURE 5:**
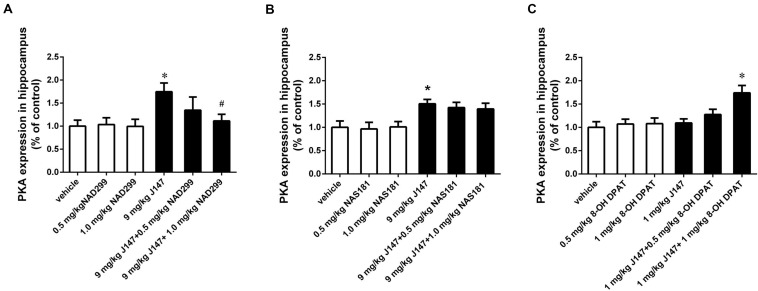
J147-induced increase in PKA level in the hippocampus was reversed by the 5-HT_1A_ receptor antagonist NAD-299, but not 5-HT_1B_ receptor antagonist NAS-181. Mice received injections of NAD-299, NAS-181, or 8-OH-DPAT, 30 min prior to J147 for 3 days. J147 at dose of 9 mg/kg increased the PKA level in the hippocampus, which was reversed by 1 mg/kg NAD-299 **(A)**, but not NAS-181 **(B)**. J147 at dose of 1 mg/kg did not affect PKA level, but its effect was potentiated by combination with 8-OH-DPAT (1 mg/kg) **(C)**. NAD-299, NAS-181 or 8-OH-DPAT used alone did not affect the PKA activity, when compared to vehicle-treated groups. The results represent the mean ± SEM, *n* = 10 per group. *p < 0.05, versus vehicle-treated group; ^#^*p* < 0.05, versus J147-treated group (9 mg/kg or 1 mg/kg).

The 5-HT_1A_ receptor antagonist NAD-299 and 5-HT_1B_ receptor antagonist NAS-181 were used for determining whether 5-HT_1A_ or 5-HT_1B_ receptor is involved in J147-induced increases in cAMP and PKA levels (Figures 4, 5). Treatment with J147 at high dose of 9 mg/kg for 3 days significantly increased cAMP levels when compared to the vehicle-treated group (*p* < 0.05). This effect was reversed by pretreatment with NAD-299 at a dose of 1.0 mg/kg for 3 days (Figure 4A; *p* < 0.05). However, pretreatment of NAS-181 for 3 days did not change the effects of J147 (9 mg/kg) on cAMP expression (Figure 4B), while 8-OH-DPAT at 1 mg/kg significantly potentiated the effects of low dose J147 (1 mg/kg) on cAMP level (*p* < 0.05; Figure 4C).

Consistently, the increased PKA levels induced by the high dose of J147 (9 mg/kg) were reversed by NAD-299 at a dose of 1.0 mg/kg (*p* < 0.05; Figure 5A). By contrast, NAS-181 did not alter the effect of J147 on PKA expression (Figure 5B). 8-OH-DPAT also significantly enhanced the sub-threshold dose of J147 (1 mg/kg) on PKA expression (*p* < 0.05; Figure 5C). Interestingly, NAD-299, NAS-181, or 8-OH-DPAT used alone did not elicit any changes in cAMP or PKA levels. These results suggested that the 5-HT_1A_ receptor, rather than the 5-HT_1B_ receptor, mediates the effects of J147 on cAMP and PKA expression.

### J147-Induced pCREB and BDNF Expression in the Hippocampus Was Reversed by 5-HT_1A_ Receptor Antagonist and Potentiated by 5-HT_1A_ Agonist

**FIGURE 6:**
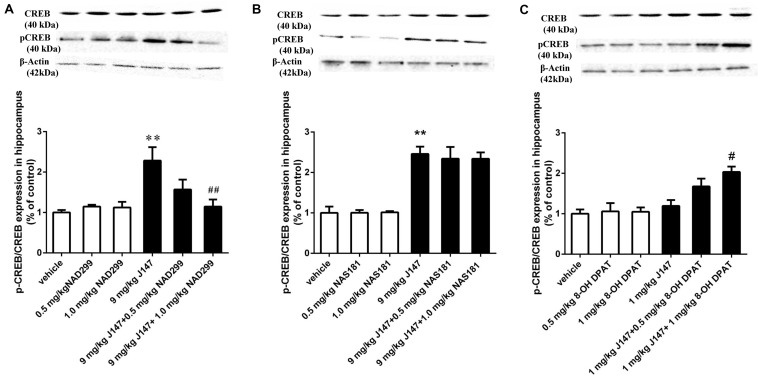
J147-induced pCREB expression in the hippocampus was reversed by 5-HT_1A_ receptor antagonist and potentiated by 5-HT_1A_ agonist. Mice received injections of NAD-299, NAS-181 or 8-OH-DPAT, 30 min prior to J147 for 3 days. J147 at dose of 9 mg/kg increased pCREB expression in the hippocampus, which was reversed by 1 mg/kg NAD-299 **(A)**, but not by NAS-181 **(B)**. J147 at dose of 1 mg/kg did not change pCREB levels, but combination with 8-OH-DPAT (1 mg/kg) potentiated such effects **(C)**. NAD-299, NAS-181, or 8-OH-DPAT used alone did not affect the pCREB expression when compared to vehicle-treated groups. The results represent the mean ± SEM, *n* = 10 per group. **p < 0.01, versus vehicle-treated groups; ^#^*p* < 0.05, ^##^*p* < 0.01, versus J147-treated group (9 mg/kg or 1 mg/kg).

To determine whether the activation of 5-HT_1A_ or 5-HT_1B_ receptor are related to the effects of J147 on the phosphorylation of CREB at Ser^133^ (pCREB) and on BDNF expression, the levels of pCREB and BDNF in the hippocampus were measured by immunoblot analysis. We found that treatment with J147 at a dose of 9 mg/kg for 3 days significantly increased pCREB at Ser^133^, when compared to vehicle-treated groups (*p* < 0.05; Figure 6A). This effect was reversed by pretreatment with NAD-299 at the dose of 1.0 mg/kg (*p* < 0.01). However, NAS-181 did not impact the increase of pCREB expression induced by J147 (9 mg/kg) (Figure 6B). Consistently, although low dose of J147 (1 mg/kg) did not induce any change in pCREB expression in the hippocampus, pretreatment of 8-OH-DPAT at 1.0 mg/kg significantly potentiated the effects of low dose J147 (*p* < 0.05; Figure 6C).

**FIGURE 7:**
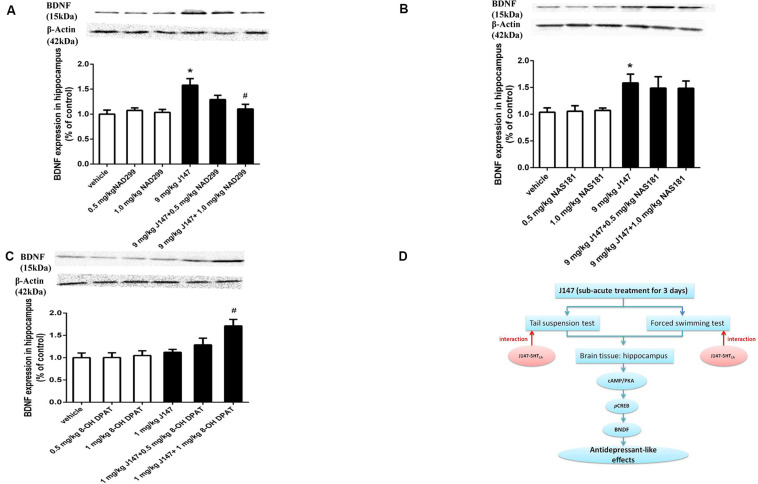
J147-induced BDNF expression in the hippocampus was reversed by 5-HT_1A_ receptor antagonist and potentiated by 5-HT_1A_ agonist. Mice received injections of NAD-299, NAS-181 or 8-OH-DPAT, 30 min prior to J147 for 3 days. J147 at dose of 9 mg/kg increased BDNF expression in the hippocampus, which was reversed by 1 mg/kg NAD-299 **(A)**, but not by NAS-181 **(B)**. J147 at dose of 1 mg/kg did not change BDNF level, but combination with 8-OH-DPAT (1 mg/kg) potentiated such effects **(C)**. The graphic representation of the proposed molecular pathway involved in the antidepressant-like effects of J147 is shown in **(D)**. NAD-299, NAS-181, or 8-OH-DPAT used alone did not affect the BDNF level when compared to vehicle groups. The results represent the mean ± S.E.M., *n* = 10 per group. ^∗^*p* < 0.05, versus vehicle-treated groups; ^#^*p* < 0.05, versus J147-treated group (9 mg/kg or 1 mg/kg).

Similar findings were observed in BDNF expression in the hippocampus. J147 at a dose of 9 mg/kg over 3 days significantly increased BDNF expression (*p* < 0.05; Figure 7A). This effect was reversed by pretreatment with NAD-299 (*p* < 0.05; Figure 7A) but not NAS-181 (Figure 7B). 8-OH-DPAT significantly potentiated sub-threshold dose of J147 at 1 mg/kg on BDNF expression (*p* < 0.05; Figure 7C). NAD-299, NAS-181 or 8-OH-DPAT used alone did not affect either the pCREB or the BDNF level when compared to vehicle-treated groups. These findings indicated that the 5-HT_1A_ receptor is involved in J147 induced pCREB and BDNF expression (Figure 7D).

## Discussion

Acute administration of J147 was previously shown to reduce the immobility time in both TST and FST tests, two classical despair models widely used as screening assays for novel antidepressants’ activities. The present study suggested that sub-acute treatment of mice with 3 mg/kg and 9 mg/kg of J147 for 3 days produced anti-immobility effects that were paralleled to those seen with the acute treatment, which suggested that acute treatment of J147 at doses of 5 and 10 mg/kg reduced immobility time both in the TST and FST (Lian et al., 2018). We did not observe any evidence indicating the development of tolerance to the drug in mice treated with J147 for 3 consecutive days. To dissociate the stimulation or inhibition of J147 on the central nervous system, we investigated the effects of different doses of J147 on mouse LMA. The results suggested that those doses administered for 3 days that affected immobility time did not induce any change in the LMA, indicating that J147 produces the specific antidepressant-like effects. Subsequently, we determined whether 5-HT receptors participated in the antidepressant-like effects of J147 by pharmacological interaction methods. The receptor binding studies demonstrated that J147 displayed high affinity for rat 5-HT_1A_ receptor and was less potent at 5-HT_1B_ receptor. Furthermore, 5-HT_1A_ receptor antagonist NAD-299 reversed J147-induced reduction of immobility time. These were supported by the fact that 5-HT_1A_ receptor agonist 8-OH-DPAT potentiated such effects. Moreover, J147 induced increases in cAMP, PKA, pCREB, and BDNF levels were reversed by NAD-299 and potentiated by 8-OH-DPAT. However, 5-HT_1B_ receptor antagonist NAS-181 did not affect J147-induced behavioral changes and downstream signaling molecules expression. These results indicate that the antidepressant-like effects of J147 are related to stimulation of 5-HT_1A_ receptor and its dependent cAMP signaling. These results indicate that in this assay, J147 eventually behaves as a 5-HT1A receptor agonist.

Depressive symptoms can be induced by depleting tryptophan, which causes a temporary reduction in central serotonin levels (Chen et al., 2011; Garcia-Garcia et al., 2014; Nautiyal and Hen, 2017). 5-HT_1A_ and 5-HT_1B_ receptors are responsible for pathological changes in depression (Perera et al., 2001; Blier and Ward, 2003). 5-HT_1A_ receptor is one of 14 known 5-HT receptor variants that gets the most attention largely owing to its key role in the pathogenesis of depression and the action of antidepressants (Lv et al., 2018). 5-HT_1B_ receptor is responsible for the dynamic accommodation of the serotonergic pathway that has been implicated in several functions such as cognition and emotion (Blier and Ward, 2003; Manuel-Apolinar and Meneses, 2004). The antidepressant-like effects of curcumin have been proven to be related to 5-HT_1A_ and 5-HT_1B_ receptors (Lv et al., 2018), as shown in our previous studies that suggested that curcumin reversed the decreases in 5-HT and 5-HT_1A_ receptor mRNA expression in chronically stressed rats (Xu et al., 2005a, 2011a). Pre-treatment with a NAD-299 or isamoltane (5-HT_1B_ receptor antagonist) abolished the neuroprotective effects of curcumin, indicating that 5-HT_1A_ and 5-HT_1B_ receptors may indeed mediate the effects of curcumin (Xu et al., 2011a, b). J147, a curcumin derivative, has neuroprotective effects in Alzheimer’s disease and in streptozotocin-induced diabetic peripheral neuropathy (Kulkarni et al., 2008; Lv et al., 2018). Acute treatment of J147 exerts antidepressant-like effects in mouse model of despair tests, mainly through serotonergic synaptic availability (Kulkarni et al., 2008; Xu et al., 2011a). The present study found that sub-acute treatment of J147 for 3 days significantly decreased the immobility time in both the FST and TST, supporting that J147 has antidepressant-like effects. To further clarify the possible mechanisms underlying its therapeutic effects, we investigated whether 5-HT_1A_ or 5-HT_1B_ receptors are involved in these effects. Our results suggested that J147 induced reduction of immobility time in the FST and TST were reversed by the 5-HT_1A_ receptor antagonist NAD-299. Moreover, the 5-HT_1A_ receptor agonist 8-OH-DPAT potentiated the effects of sub-threshold dose of J147 on depression-like behaviors. The 5-HT_1B_ receptor antagonist NAS-181 did not reverse high dose J147-induced reduction of immobility time in either the FST or the TST. These results suggested that 5-HT_1A_ receptor might participate in the antidepressant-like effect of sub-acute treatment of J147, which is consistent with our previous study (Nautiyal and Hen, 2017).

Some clinical and preclinical studies indicated that traditional antidepressants stimulate cAMP signaling and produce antidepressant-like effects (Xu et al., 2011b). A current study demonstrated that J147 could bind to mitochondrial α-F1 subunit of ATP synthase (ATP5A) and partially regulated the activity of the mitochondrial ATP synthase (Goldberg et al., 2018), which indicated that J147 might exert its effects by regulating cAMP levels. Usually, antidepressant agents activate the cAMP pathway including activation of PKA, phosphorylation and activation of CREB, and the direct stimulation of its downstream target BDNF (Duman et al., 1997; Xu et al., 2005b). The BDNF gene contains a cAMP response element (CRE), to which phosphorylated CREB binds and thereby in turn enhance CREB transcription (Zhang et al., 2016). Knockout of BDNF in the hippocampus or in the prefrontal cortex blocked the antidepressant-like effects of SSRIs (Zhang et al., 2016). However, whether J147 induces BDNF expression directly or via 5-HT_1A_ receptor mediated pathway is still unknown. Although some studies demonstrate that 5-HT_1A_ is coupling via Gi/Go proteins to inhibit cAMP, other studies including ours describe that cAMP is activated by 5-HT_1A_ receptor, which induces PKA expression and downstream CREB phosphorylation and BDNF expression (Xu et al., 2011b; Lian et al., 2018; Qiu et al., 2018). The 5-HT_7_ receptor is one of the members of the 5-HT receptor family (Barnes and Sharp, 1999; Hedlund and Sutcliffe, 2004), which stimulates cAMP formation by activating AC via the Gs proteins (Norum et al., 2003). Indeed, 5-HT_7_ receptors are known to form heterodimers with 5-HT_1A_ receptors (Renner et al., 2012) and this heterodimer modulates cAMP production. Our present study suggested that J147 increased 5-HT_1A_ and 5-HT_7_ receptor levels, which indicate that J147 may stimulate the interaction of 5-HT_1A_ and 5-HT_7_ and upregulate the cAMP levels. These results were supported by the subsequent findings that demonstrated that sub-acute treatment with J147 increased cAMP, PKA, pCREB, and BDNF expression in the hippocampus of mice. NAD-299, rather than NAS-181, reversed the effects of J147 on these proteins’ expression in the hippocampus. 8-OH-DPAT potentiated the effects of low dose J147 on the aforementioned proteins’ expression. These results were consistent with our behavioral findings and further supported our hypothesis, which indicate that the antidepressant-like effects of J147 are mediated by the 5-HT_1A_ receptor-cAMP-PKA-pCREB-BDNF related signaling pathway in the hippocampus.

In summary, the present results suggest that sub-acute treatment of J147 produces antidepressant-like effects in the mouse model of despair tests, particularly the TST and FST. Moreover, sub-acute treatment of J147 did not result in reduction of therapeutic effect or development of drug tolerance. These findings demonstrate that J147 could be a novel antidepressant agent.

## Data Availability Statement

All datasets presented in this study are included in the article/Supplementary Material.

## Ethics Statement

The animal study was reviewed and approved by The Animal Care and Use Committee of the State University of New York at Buffalo.

## Author Contributions

YY and YX conceived and designed the study, provided critical comments and edited the manuscripts. JLi, LC, and GL performed experiments on mice including the acquisition, analysis and interpretation of data. XC and SH performed the analysis and interpretation of data on immunoblot analysis and Elisa kit assay. LZ performed on data collecting. YS drafted and revised the manuscript. VL and JLv edited the manuscript. All authors read and approved the final manuscript.

## Conflict of Interest

The authors declare that the research was conducted in the absence of any commercial or financial relationships that could be construed as a potential conflict of interest.
