# Supplementary material for: Sub-Acute Treatment of Curcumin Derivative J147 Ameliorates Depression-Like Behavior Through 5-HT1A-Mediated cAMP Signaling
Source: Front Neurosci. 2020 Jul 8;14:701. doi: 10.3389/fnins.2020.00701 (PMC7360862; doi:10.3389/fnins.2020.00701)
Supplement: Supplementary file 1 [file Table_1.DOCX]

**
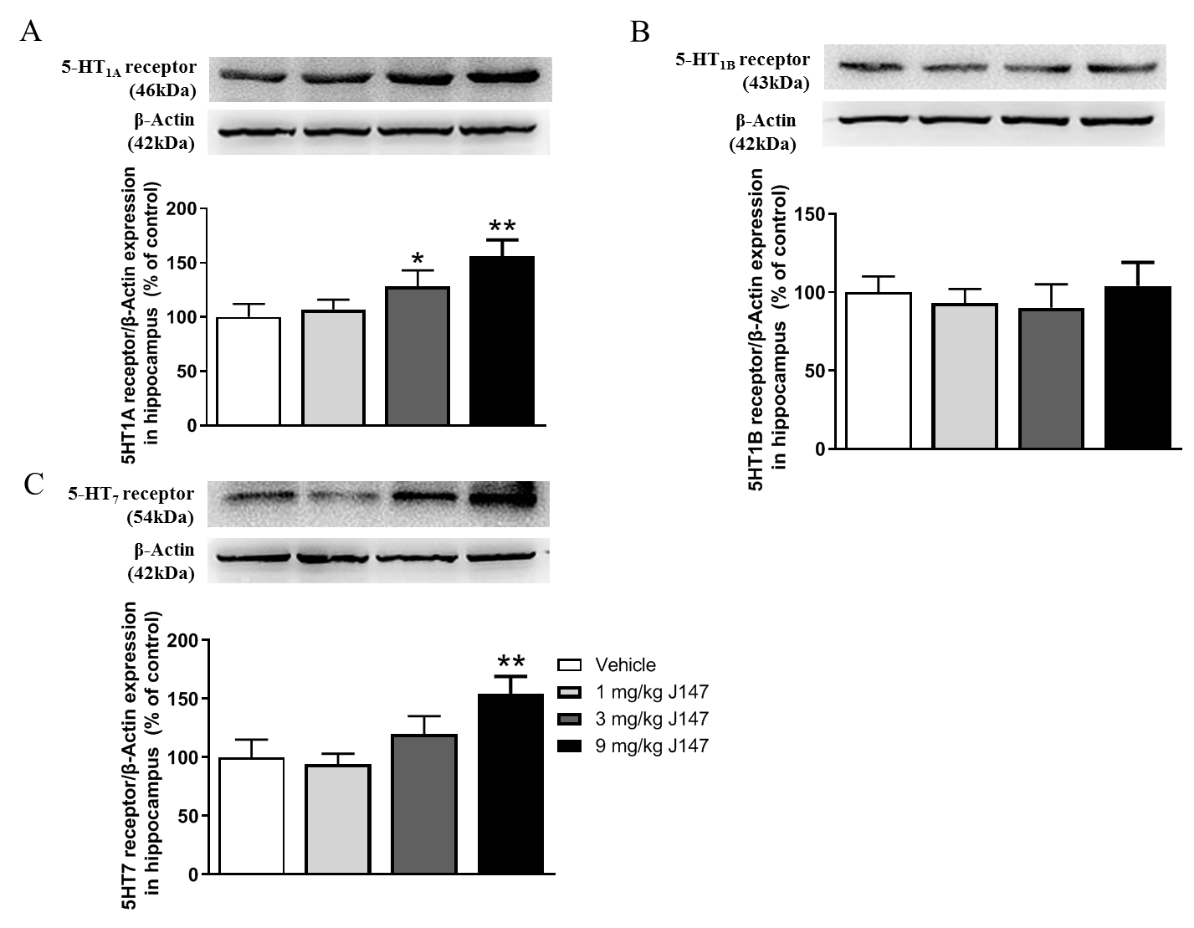
**

**Supplementary Fig. 1** The effects of J147 on 5-HT_1A_, 5-HT_1B_ and 5-HT_7_ receptors expression in the hippocampus. The results represent the mean ± S.E.M., n = 10 per group. ^*^*p*<0.05, ^* *^ *p*<0.01 vs. vehicle-treated group.
